# Establishment of a Dual-Vector System for Gene Delivery Utilizing Prototype Foamy Virus

**DOI:** 10.4014/jmb.2312.12026

**Published:** 2024-02-15

**Authors:** Soo-Yeon Cho, Yoon Jae Lee, Seong-Mook Jung, Young Min Son, Cha-Gyun Shin, Eui Tae Kim, Kyoung-Dong Kim

**Affiliations:** 1 Department of Systems Biotechnology, Chung-Ang University, Anseong 17456, Republic of Korea; 2 Department of Microbiology and Immunology, Jeju National University College of Medicine, Jeju 63241, Republic of Korea; 3 Department of Biomedicine & Drug Development, Jeju National University, Jeju 63241, Republic of Korea

**Keywords:** Foamy viral vector, platform study, gene therapy, codon-optimized Env

## Abstract

Foamy viruses (FVs) are generally recognized as non-pathogenic, often causing asymptomatic or mild symptoms in infections. Leveraging these unique characteristics, FV vectors hold significant promise for applications in gene therapy. This study introduces a novel platform technology using a pseudo-virus with single-round infectivity. In contrast to previous vector approaches, we developed a technique employing only two vectors, pcHFV lacking Env and pCMV-Env, to introduce the desired genes into target cells. Our investigation demonstrated the efficacy of the prototype foamy virus (PFV) dual-vector system in producing viruses and delivering transgenes into host cells. To optimize viral production, we incorporated the codon-optimized Env (optEnv) gene in pCMV-Env and the Woodchuck Hepatitis Virus Posttranscriptional Regulatory Element (WPRE) at the 3’ end of the transgene in the transfer vector. Consequently, the use of optEnv led to a significant enhancement in transgene expression in host cells. Additionally, the WPRE exhibited an enhancing effect. Furthermore, the introduced EGFP transgene was present in host cells for a month. In an effort to expand transgene capacity, we further streamlined the viral vector, anticipating the delivery of approximately 4.3 kbp of genes through our PFV dual-vector system. This study underscores the potential of PFVs as an alternative to lentiviruses or other retroviruses in the realm of gene therapy.

## Introduction

Over the past three decades, viral vectors have played a role in more than 2,000 clinical interventions, encompassing a wide range of applications from therapeutic treatments to regenerative medicine, with more than half advancing to phase I clinical trials according to the database of the Journal of Gene Medicine (https://www.abedia.com/wiley/index.html). The gene therapy field has achieved notable clinical successes and regulatory approval based on key strategies using viral vectors from adeno associated viruses, adenoviruses, and lentiviruses. Nonetheless, it still faces several challenges, especially the safety concerns, including unwanted immune responses and infections caused by the viruses [1-3].

Retroviruses can be classified into two subfamilies: Orthoretrovirinae, including the genera Lentivirus and Gammaretrovirus, and Spumaretrovirinae, including foamy viruses [4]. Since the isolation of ‘foamy viral agent’ in 1955, FVs have been detected in various mammals, including humans [5]. The best characterized FV isolate is the prototype FV (PFV), which was obtained from an infected Kenyan individual who was probably infected by trans-species infection with simian foamy virus (SFV) [6]. FVs are exogenous viruses that elicit a distinct cytopathic effect (CPE), or a characteristic ‘foamy’ appearance in host cells [7]. Despite their ability to cause this effect, FVs are generally considered non-pathogenic, with infections typically being asymptomatic or result in only mild symptoms [8, 9]. FV vectors exhibit a preference for integration near regulatory elements such as CpG islands, whereas lentiviral vectors have a tendency to integrate within coding sequences [8-11]. These distinctive features of FVs make their vectors highly promising in gene therapy.

Research on foamy viral vectors is still in ongoing. Historically, the primary focus has been on animals, particularly canines and felines [4, 12-14]. FV vectors can transduce several cell lines, murine hematopoietic progenitors, and stem cells [15-17]. FV exhibits a wide-ranging tropism for target cells by interacting with heparan sulfate, a prevalent membrane-bound macromolecules [18, 19]. Given the broad infectivity of the FV, even in humans, its future potential as a next-generation vector is contingent on establishing its stability and high transduction rates into cells [5].

In this context, we introduce a novel PFV dual-vector system that generates defective PFVs designed to infect host cells only once with only two vectors, pcHFV lacking Env and pCMV-Env. We demonstrate the effective infection capability of these PFVs and the sustained expression of the EGFP transgene in host cells. Our findings underscore the potential of PFVs as a viable alternative to lentiviruses or other retroviruses in gene therapy.

## Materials and Methods

### Plasmid Construction

For the construction of EGFP transgene vector (pcHFV-EF1a-EGFP_v1, 2, and 3), an infectious molecular clone of primate FV containing a cytomegalovirus intermediate-early promoter (pcHFV) was used as the backbone vector. The pcHFV vector was linearized with *Blp*I and *Bsp*EI, followed by assembly with EF1a core promoter and EGFP sequence using EZ-Fusion HT Cloning Kit (Enzynomics, Republic of Korea). The WPRE sequence was inserted downstream of the EGFP sequence in the same manner. For the Env-expressing vector (pCMV-Env), the PFV *Env* gene was substituted for the EGFP sequence in the pEGFP-C3 backbone vector using standard cloning techniques. To create a codon-optimized Env-expressing vector, the full-length PFV *Env* gene with human codons was synthesized and cloned into the pEGFP-C3 vector using gene synthesis and cloning services provided by GenScript (USA).

### Cell Culture

Human fibrosarcoma cell line HT1080 was obtained from the Korean Cell Line Bank, and the human embryonic kidney cell line 293FT was obtained from Invitrogen (Thermo Fisher Scientific, USA). All cells were grown in Dulbecco’s modified Eagle’s medium (DMEM), supplemented with 10% fetal bovine serum (FBS) and 1%streptomycin/penicillin, and cultured in a humidified 5% CO_2_ incubator at 37°C. For the cell viability assay, transduced HT1080 cells were sub-cultured at 1:10 or 1:8 dilution every 3 to 4 days for up to 28 days.

### Virus Production and Infection

All transfection procedures were carried out using jetOPTIMUS (Polyplus, Germany), following the manufacturer's protocol. 293FT cells were cultured in a 6-well plate until reaching at 70-80% confluency and transfected with a total of 3 μg DNA, comprising both the EGFP transgene vector and the Env-expressing vector. Culture supernatants were collected 2-3 days post-transfection and subsequently filtered through a 0.45 μm syringe filter. For pseudo-virus containing the EGFP gene cassette (PFV-EGFP) transduction, 1 ml of the virus-containing supernatants were treated to -1.0 × 10^6^ of HT1080 cells per well in a 6-well plate, repeated three times with intervals of 2-2.5 h.

### Determination of the Virus Titer

The PFV-EGFP titer was assessed by calculating the percentage of GFP-positive cells among the infected cells at 3 days post-infection using flow cytometry. The formula employed for measuring the viral titer is as follows: Transduction Unit per ml (TU/ml) = [(F x Cn) / V] x DF. Here, F represents the frequency of GFP-positive cells as determined by flow cytometry, Cn is the total number of cells utilized for transduction, V is the volume of the treated culture supernatants, and DF denotes the virus dilution factor.

### Fluorescence Microscopy

Fluorescence microscopy was performed using a inverted microscope Axio Observer 3 (Zeiss, USA) equipped with a excitation wavelength 475 nm to visualize the EGFP signal. Image was captured using a Axiocam 506 mono, and image analysis was conducted using a Zen 3.0 software for visualization and analysis.

### Flow Cytometry

After 3-4 days post-transduction, cells were harvested and resuspended in 100 μl of 1X PBS buffer per sample. Resuspended cells were treated with Zombie Violet Fixable Viability dye (1:500 dilution, BioLegend, USA) in the dark at 4°C for 30 min. Following staining, cells were washed with 1X PBS buffer through centrifugation at 1500 rpm, 4°C for 5 min and then resuspended in 500 μl of ice-cold FACS buffer [1X PBS (pH 7.2) with 2% FBS, 2 mM EDTA, and 0.09% sodium azide]. Flow cytometry analysis was performed using the Attune NxT Flow Cytometer (Thermo Fisher Scientific) and FlowJo software (Treestar, USA).

### Western Blotting

Cells were lysed in RIPA lysis buffer [150 mM NaCl, 1% Non-ionic detergent, 1% Sodium deoxycholate, 0.1%SDS, (Biomax, Republic of Korea)]. About 20 μg of proteins per sample were separated by SDS-PAGE using a 10%polyacrylamide gels and transferred to nitrocellulose membranes (Cytiva, USA). Membranes were blocked with 10% (w/v) non-fat dry milk in TBST buffer [100 mM Tris (pH 7.6), 150 mM NaCl, 0.1% (w/v) Tween-20] for 1 h at room temperature. Membranes were incubated with a 1:1,000 dilution of rabbit polyclonal anti-Gag antibody, 1:500 dilution of rabbit polyclonal anti-Env antibody, 1:5,000 dilution of rabbit monoclonal GFP antibody (Abcam, UK), and 1:5,000 dilution of rabbit monoclonal β-actin antibody (Cell Signaling Technology, USA) at 4°C overnight. The membranes were then incubated with a 1:10,000 dilution of secondary antibody, goat anti-rabbit IgG conjugated to HRP (BioRad, USA), for 1 h at room temperature. The chemiluminescent signal was visualized by exposing the blots to a chemiluminescence imaging system, E-blot (e-BLOT Life Science, China). Rabbit polyclonal anti-Env antibody was designed to be specific for PFV Env-SU domain and was custom-produced by AbClon (Korea).

### Statistical Analysis

Data in this study were presented as the mean ± SEM (Standard Error of Mean) of more than three independent experiments. Statistical significance (*p*-value) was determined by the Student’s *t*-test (*** *p* < 0.001, ** *p* < 0.01, * *p* < 0.05, ns p > 0.05).

## Result

### Generation of the PFV Dual-Vector System

**Fig. 1:**
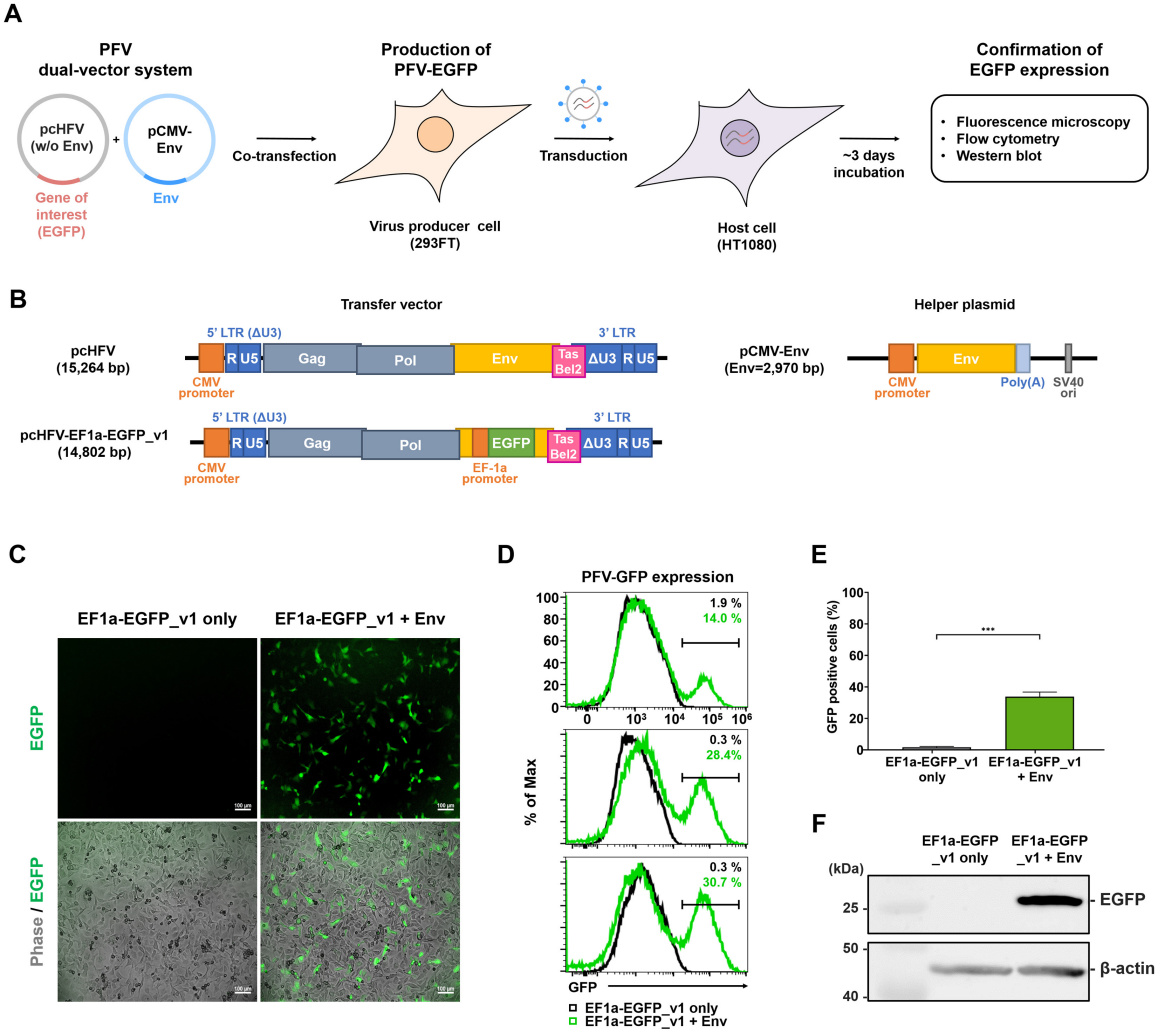
Expression of *EGFP* transgene in HT1080 cells using the PFV dual-vector system. (**A**) The process involves the production and transduction workflow of PFV-EGFP. 293FT cells were co-transfected with an EGFP transgene vector (pcHFV lacking Env) and a PFV Env-expressing vector (pCMV-Env). The virus-containing supernatant was applied to HT1080 cells for transduction. After 3 days of infection, the expression of EGFP was verified through fluorescence microscopy, flow cytometry, and western blot analysis. (**B**) A schematic diagram illustrating the PFV proviral vector (pcHFV), PFV proviral vector with EGFP transgene controlled by the EF1a core promoter (pcHFV-EF1a-EGFP_v1), and Env-expressing vectors (pCMV-Env). (**C-F**) HT1080 cells were transduced with PFV produced by pcHFV-EF1a-EGFP_v1 and pCMV-Env at a 2:1 weight ratio. At 3-4 days post-infection (dpi), the expression of the EGFP transgene delivered via PFV-EGFP was confirmed by fluorescence microscopy (**C**), flow cytometry (**D, E**), and western blot (**F**). Value represent mean ± SEM of more than three independent experiments (**E**). HT1080 cells treated with supernatant from 293FT cells transfected only with pcHFV-EF1a- EGFP_v1 were used as a negative control.

We established the PFV dual-vector system, which comprises the pcHFV with a partially deleted *Env* fragment (8,644-9,091 nt) and pCMV-Env (Fig. 1A). In this setup, the Env encoding gene was replaced with a transgene, such as EGFP, regulated by EF-1a internal promoter (pcHFV-EF1a-EGFP_v1) (Fig. 1B). Notably, the absence of an internal promoter led to a significant reduction in the transgene expression rate (data not shown). Env was introduced via an independent expression vector, pCMV-Env (Fig. 1A and 1B). Co-transfection of these two vectors into 293FT cells resulted in the production of PFV incorporating EGFP (PFV-EGFP). Subsequently, viral supernatants were collected and applied to HT1080 cells to assess the efficacy of transgene expression. Monitoring of EGFP expression was performed using fluorescence microscopy, flow cytometry, and western blot analysis (Fig. 1A).

We observed successful generation of PFV virus with EGFP expression in HT1080 cells through co-transfection of pcHFV-EF1a-EGFP_v1 and pCMV-Env at a 2:1 weight ratio (total 3 μg), as confirmed by fluorescence microscopy (Fig. 1C). We explored various weight ratios between pcHFV-EF1a-EGFP_v1 and pCMV-Env to find the optimal ratio while maintaining a constant total DNA amount of 3 μg throughout all experiments. Based on our pilot experiments, the 2:1 weight ratio yielded the most effective viral infection efficacy (data not shown). Flow cytometry analysis from three independent experiments revealed average rates of EGFP-positive cells at 14.0%, 28.4%, and 30.7% (Fig. 1D and 1E). Furthermore, the presence of EGFP was validated using an anti-GFP antibody (Fig. 1F). Taken together, our PFV dual-vector system effectively produces PFV and facilitates the expression of foreign genes in host cells.

### Introduction of Codon-Optimized Env Sequence and WPRE

**Fig. 2:**
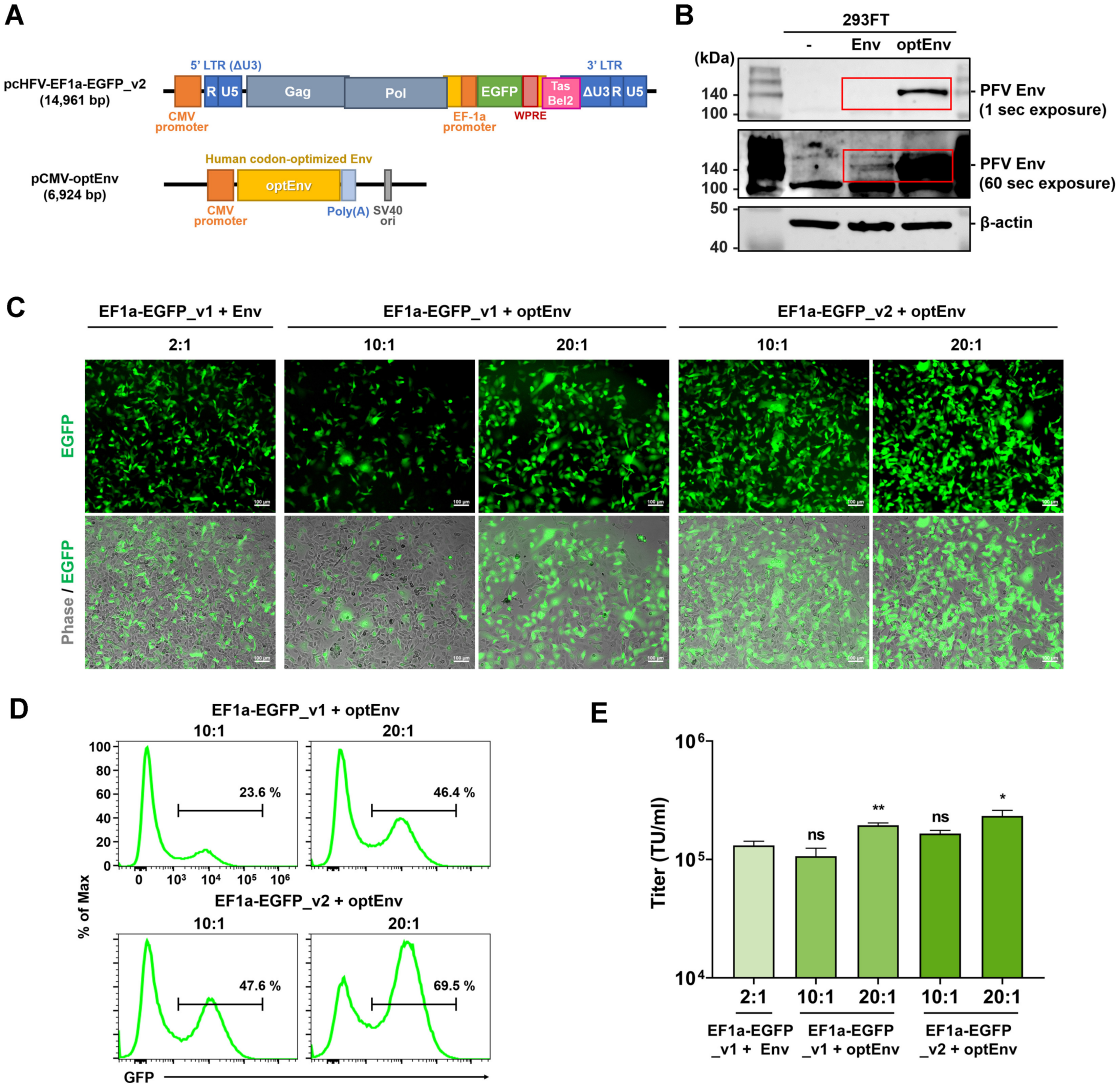
EGFP transgene expression from the codon optimized Env protein and WPRE. (**A**) Schematic representation of human codon-optimized Env-expressing vectors (pCMV-Env codon-optimized: optEnv) and the EGFP transgene vectors with WPRE (pcHFV-EF1a-EGFP_v2) used in this study. (**B**) Western blot analysis depicting the expression of original Env and optEnv with b-actin control in 293FT cells. (**C-E**) Generation of PFV-EGFP by co-transfecting pcHFVEF1a- EGFP_v1/v2 and Env-expressing vectors at different ratios as indicated. The impact of optEnv and the WPRE was validated using fluorescence microscopy (**C**) and flow cytometry (**D, E**) at 3 dpi. Value represent mean ± SEM of more than three independent experiments (**E**).

In order to enhance the efficiency of the infection rate, we synthesized codon-optimized Env (optEnv) sequence and substitute it for the original Env sequence on pCMV-Env (Fig. 2A). The western blot analysis with a custom anti-Env antibody confirmed that the expression of optEnv protein was significantly more stable than that of the intact Env in transfected 293FT cells (Fig. 2B).

Based on our pilot experiment, around 20:1 weight ratio (total 3 μg) of pcHFV-EF1a-EGFP and pCMV-optEnv resulted in the most effective viral infection efficacy, likely due to the more stable expression of optEnv compared to the original Env (Fig. 2C). Consequently, we proceeded to compare the viral infection rates of different sample set under their respective optimal conditions, 2:1 for the original Env and 10:1 or 20:1 for optEnv. We noticed that optEnv compared to the original Env yielded better viral infection efficacy, resulting in an increased rate of EGFP expression at a 20:1 weight ratio of EF1a-EGFP and optEnv (Fig. 2C). Flow cytometry analyses revealed that optEnv resulted in a substantial increase in EGFP-positive cell populations, reaching up to 46.4% at a 20:1 weight ratio (Fig. 2D). The average scores of EGFP-positive cells were 44.4% (2X10^5^ TU/ml) at a 20:1 ratio of EF1a-EGFP and optEnv (Fig. 2E).

We also employed the WPRE sequence at the 3’-UTR of EGFP gene (pcHFV-EF1a-EGFP_v2). WPRE is recognized for its ability to facilitate mRNA export form the nucleus to the cytoplasm, thereby augmenting the expression of the target gene [20]. Interestingly, we detected an additive effect of the WPRE on EGFP expression, leading to a general increase in EGFP expression compared to when the WPRE element is absent. This suggests that WPRE may enhance the expression of target genes.

### PFV Vectors Enable Stable Transgene Expression without Progeny Production

**Fig. 3:**
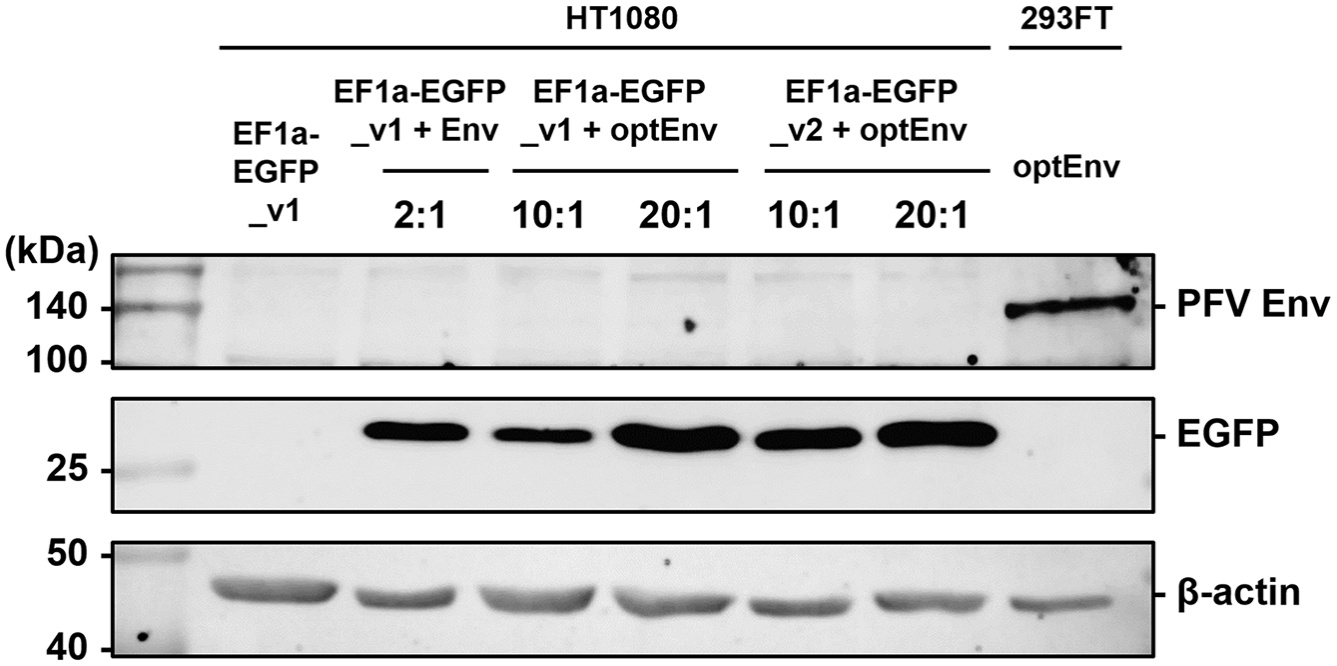
Lack of Env expression in HT1080 cells upon re-infection. Western blot analysis investigating the presence of PFV Env proteins and EGFP proteins in HT1080 cells at 3 dpi. The β-actin was used as a loading control.

To assess the absence of any supplementary virus production resulting from PFV vector infection, we investigated the expression of Env proteins in HT1080 cells post-transduction. Given that our PFV dual-vector systems incorporate a defective Env from pcHFV vector, Env expression is anticipated to be absent, thereby preventing any further virus production. Western blot analysis corroborated the absence of Env protein detection, whereas EGFP proteins were successfully expressed in HT1080 cells post-transduction (Fig. 3). Therefore, our PFV dual-vector systems does not lead to any additional production of infectious viruses in HT1080 cells following transduction.

**Fig. 4:**
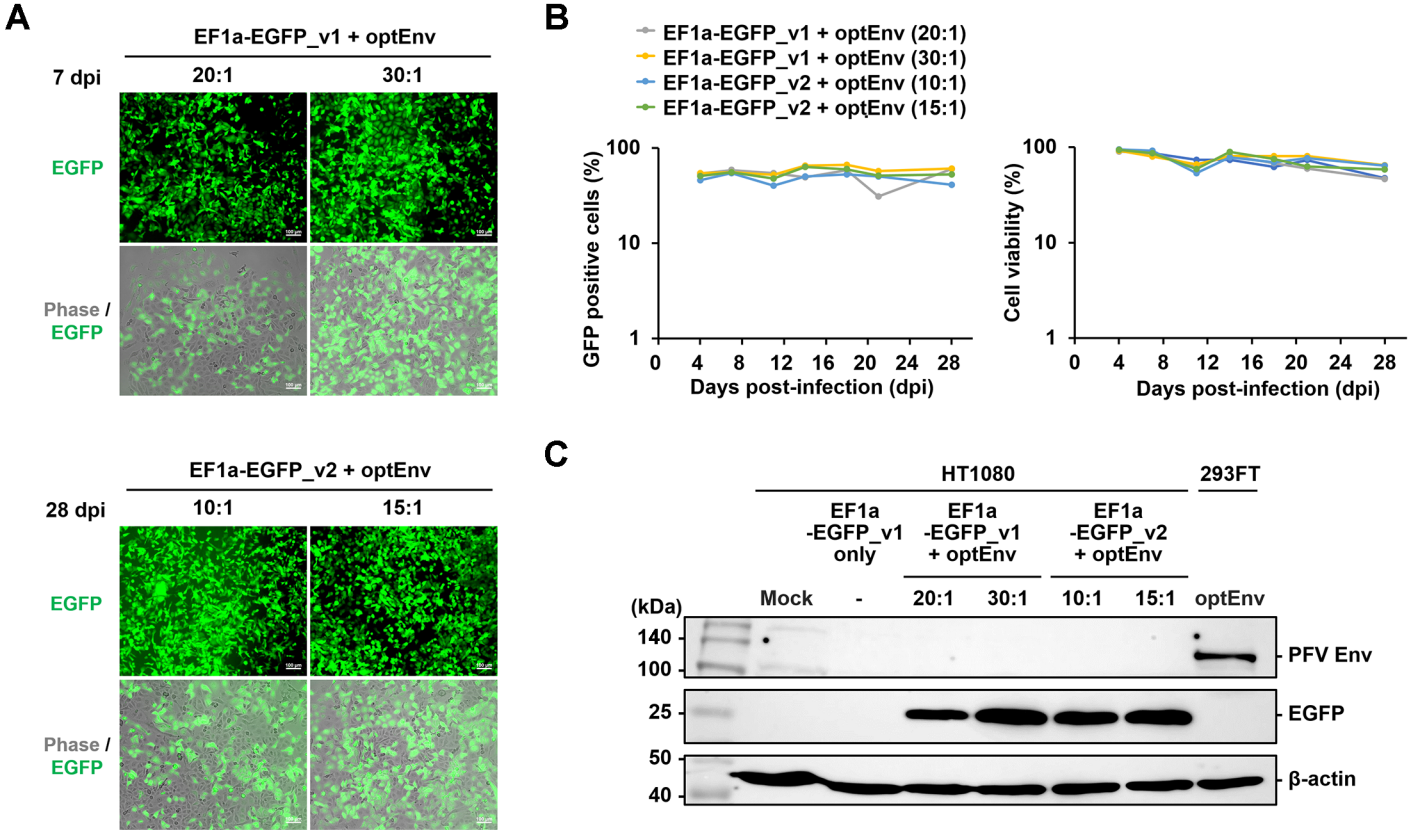
Prolonged expression of EGFP gene in HT1080 cells. (**A**) Fluorescence microscopy images depicting the expression of PFV-EGFP in HT1080 cells under the specified conditions at day 7 and day 28 post-infection. (**B**) Flow cytometry analysis monitoring the expression of PFV-EGFP and cell viability from day 4 to day 28 post-infection. (**C**) Western blot analysis showing the expression of PFV Env, PFV Gag, and EGFP, with β-actin as a control, in HT1080 cells at day 28 postinfection. The HT1080 cell line served as the Mock, and optEnv-transfected 293FT cells were used as a negative control.

Next, we investigated the enduring expression of EGFP proteins in HT1080 cells following transduction using fluorescence microscope and flow cytometry analysis (Fig. 4A and 4B). Under both conditions, EF1a-EGFP_v1 plus optEnv and EF1a-EGFP_v2 plus optEnv, the introduced EGFP gene exhibited consistent and stable expression from day 4 to day 28. By day 28, the percentage of EGFP-positive cells had reached 60.0% (Fig. 4A and 4B). However, cell viability showed a gradual decline over time, potentially attributed to the constitutive expression of the EGFP protein or technical issues during cell culture passaging (Fig. 4B). Western blot analysis further confirmed the sustained expression of EGFP proteins until day 28 (Fig. 4C). Hence, our PFV dual vector system may enable the stable expression of transgenes in host cells.

### Enhancing Transgene Insertion Space in a PFV Dual-Vector System

**Fig. 5:**
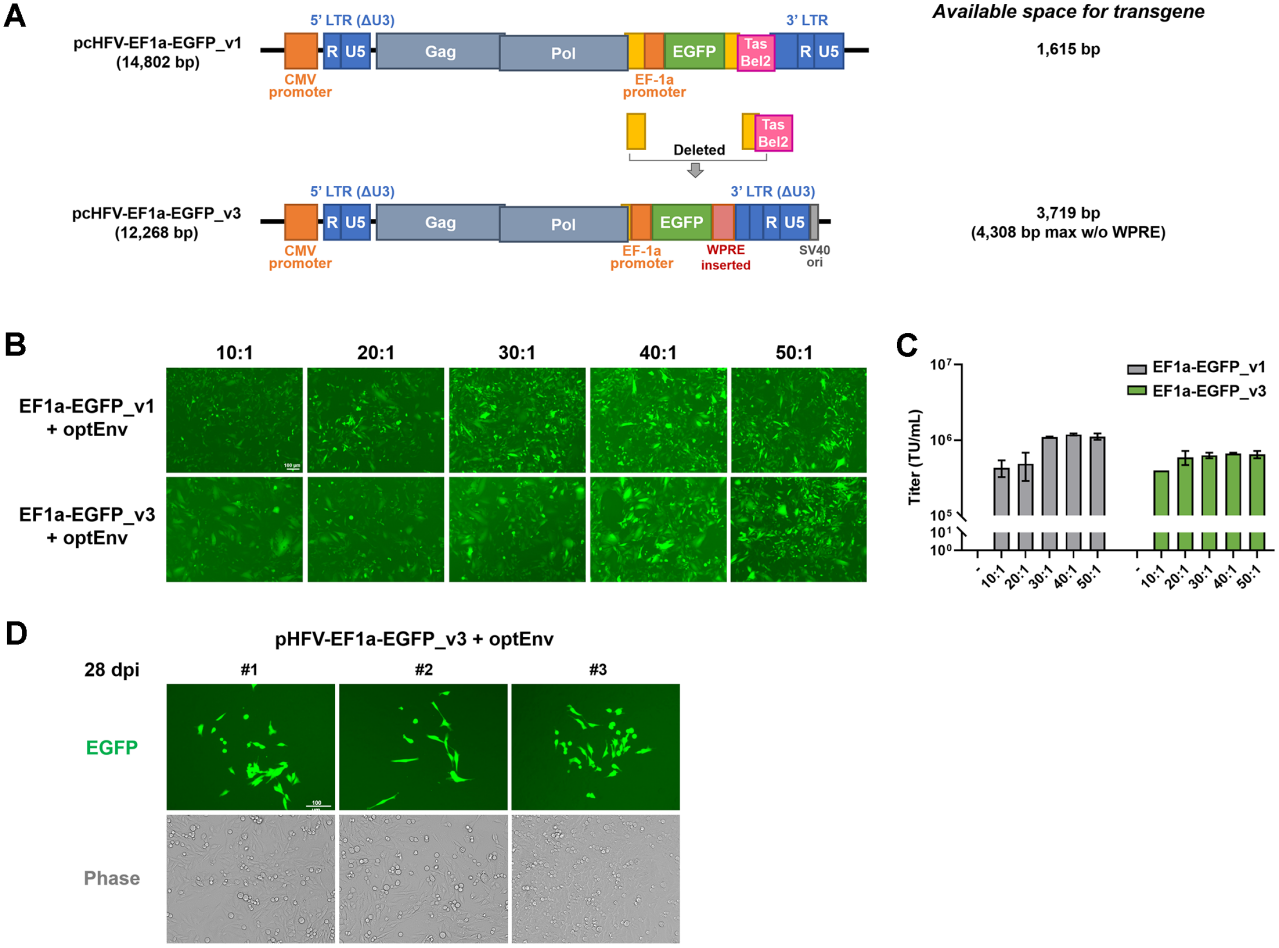
Expansion of transgene insertion space in the transfer vector. (**A**) Schematic diagram illustrating the pcHFVEF1a-EGFP plasmid with the *Env*, *Tas*, and *Bel2* gene regions removed to increase transgene insertion space. (**B**) The expression of PFV-EGFP in HT1080 cells, generated by the indicated conditions, was observed through fluorescence microscopy at 48 h post-infection. (**C**) Titers were determined as transducing units per milliliter (TU/ml). Value represent mean ± SEM of more than three independent experiments. (**D**) The expression of EGFP from transduced cells with PFV-EGFP was observed through fluorescence microscopy at 28 dpi.

The transgene insertion capacity of earlier versions (pcHFV-EF1a-EGFP_v1 and v2) was limited to a range between 1,026 bp and 1,615 bp, which significantly restricted their usability in gene therapy applications. To overcome this constraint, we systematically designed PFV vectors to accommodate larger transgene sequences. We excised the *Env* gene, except for the part overlapping with the Pol gene on the 5' side, and removed the *Tas* and *Bel2* regions on the 3' side (7,642-11,409 nt). As a result, pcHFV-EF1a-EGFP_v3 has secured a space capable of accommodating a maximum of 4,308 bp of transgene insertion (Fig. 5A). Although concerns initially arose regarding the functional integrity of the vector after these modifications, given the deletion of important regulatory genes such as *Tas* and *Bel2*, which are essential for viral replication and transcriptional regulation, our experiments demonstrated the functionality of the modified dual-vector system. Despite lacking the *Env*, *Tas*, and *Bel2* regions, the redesigned vector (pcHFV-EF1a-EGFP_v3) effectively facilitated gene delivery and expression in target cells. The new version of the vector exhibited similar performance to pcHFV-EF1a-EGFP_v1 under all conditions when measuring the transduction efficiency of progeny virions produced under various co-transfection ratios of optEnv (Fig. 5B and 5C). This achievement was the result of meticulous vector redesign, preserving essential regulatory elements while maximizing space for the transgene through the removal of *Env*, *Tas*, *Bel2*, etc. In addition, this redesigned vector maintained long-term transgene expression, similar to previous versions of the vector (Fig. 5D).

## Discussion

In this study, we have developed convenient and useful foamy virus vectors for gene delivery using a dual-vector system. Our approach centered on the removal of the *Env*, *Tas*, and *Bel2* regions, coupled with the EF1a core promoter to facilitate transgene expression. A notable aspect of this study is the separate helper plasmid used to produce the virus, particularly the Env protein. This protein underwent human codon optimization, enhancing expression levels and boosting virus production, which is a key step in maximizing the efficiency of our vector system.

Foamy virus-based vectors have been developed using a four-vector system [21-24], primarily to eliminate the risks of virus reproduction by removing the *Tas* gene [25]. This system offers the advantage of providing maximum capacity for transgene loading. However, vector engineering using four plasmids can present challenges in actual production phases, including high complexity, low efficiency, scalability limitations, and issues in cost and quality control issues. Our dual-vector system addresses these challenges by eliminating the risks associated with foamy virus vector reproduction and optimizing efficiency. Despite a limitation in transgene loading space of 4.3 kbp (Fig. 5A), our platform can accommodate a considerable number of human genes and offers the advantage of easier handling. One of the key findings of our study is the successful implementation of a dual-vector system for gene delivery. By removing fragments in the prototype virus but unnecessary in our artificial construct, we were able to simplify the vector and improve its functionality. Even without these important regulatory genes, the redesigned vector effectively promoted transgene delivery and expression in target cells. This demonstrates the robustness and efficiency of the dual-vector system.

Furthermore, the utilization of the EF1a core promoter played a crucial role in maximizing transgene expression. The EF1a promoter is known for its strong and ubiquitous activity, making it an ideal choice for driving transgene expression in a wide range of cell types [26]. By employing this promoter, we achieved sustained transgene expression in transduced cells for nearly a month without viral replication. This is a significant advantage because it addresses a major concern in gene therapy - the safety and stability of the vector system. By ensuring that the virus does not replicate in the transduced cells, we mitigate potential risks such as insertional mutagenesis or unwanted immune responses, which are commonly encountered challenges in viral vector-based gene therapies. We have also engineered the introduction of the SV40 origin sequence to enable replication of both the transfer and helper plasmids in the transfected cell lines during virus production (Figs. 2A and 5A) [27]. Although the role of the SV40 origin was not distinctly observed in small-scale pilot experiments, it is anticipated to yield a series of benefits in large-scale production (Fig. 5C).

Looking ahead, further research is warranted to explore the full therapeutic potential of our vector system. Investigations into its applicability across different cell types, long-term stability in vivo, and its scalability for clinical use are essential next steps. In addition, comparative studies with other viral vector systems could provide deeper insights into the unique advantages and limitations of our foamy virus dual-vector system. By comparing our system to existing methods, we can better understand its strengths and identify areas for further improvement. Through this study, we demonstrated the feasibility of using the foamy virus vector as a simple, efficient, and safe gene delivery platform. The dual-vector system we designed expands the options for developing viral gene therapies by providing additional avenues for treating a variety of genetic diseases.
